# Chewing Analysis by Means of Electromagnetic Articulography: Current Developments and New Possibilities

**DOI:** 10.3390/s23239511

**Published:** 2023-11-30

**Authors:** Franco Marinelli, Camila Venegas, Josefa Alarcón, Pablo Navarro, Ramón Fuentes

**Affiliations:** 1 Research Centre in Dental Sciences (CICO-UFRO), Dental School—Facultad de Odontología, Universidad de La Frontera, Temuco 4780000, Chile; franco.marinelli@ufrontera.cl (F.M.); camilabelen.venegas@ufrontera.cl (C.V.); josefa.alarcon@ufrontera.cl (J.A.); pablo.navarro@ufrontera.cl (P.N.); 2 Doctoral Program in Morphological Sciences, Dental School, Universidad de La Frontera, Temuco 4780000, Chile; 3 Facultad de Ciencias de la Salud, Universidad Autónoma de Chile, Temuco 4780000, Chile; 4 Department of Integral Adults Dentistry, Dental School—Facultad de Odontología, Universidad de La Frontera, Temuco 4780000, Chile

**Keywords:** electromagnetic articulography, chewing, border movements, Posselt’s polygon, chewing cycles

## Abstract

Chewing is a complex procedure that involves sensory feedback and motor impulses controlled by the trigeminal system in the brainstem. The analysis of mandibular movement is a first approximation to understanding these mechanisms. Several recording methods have been tested to achieve this. Video, ultrasound, the use of external markers and kinesiographs are examples of recording systems used in research. Electromagnetic articulography is an alternative method to those previously mentioned. It consists of the use of electromagnetic fields and receiver coils. The receiver coils are placed on the points of interest and the 3D coordinates of movement are saved in binary files. In the Oral Physiology Laboratory of the Dental Sciences Research Center (Centro de Investigación en Ciencias Odontológicas—CICO), in the Faculty of Dentistry at the Universidad de La Frontera (Temuco, Chile) several research studies have been carried out using the AG501 3D EMA articulograph (Carstens Medizinelektronik, Lenglern, Germany). With this device, they developed a series of protocols to record mandibular movement and obtain new information, such as the 3D Posselt polygon, the area of each polygon, individualized masticatory cycles and speed and acceleration profiles. Other investigations have analyzed these parameters, but separately. The AG501 allows for holistic analysis of all these data without altering natural movement. A limitation of this technology is the interference generated by its metallic elements. The aim of the present work is to show the developed methods used to record mandibular movement in the CICO, using the AG501 and compare them with others used in several research studies.

## 1. Introduction

Mastication is a physiological process in which the food introduced into the mouth is turned into an alimentary bolus [1]. During mastication, rhythmic activity of the muscles that open and close the mouth is produced in the brainstem [2]. A system of sensory feedback, formed using a variety of muscular, intraoral and articular receptors, interacts with the central system to adapt mandible movement to the food characteristics [3,4]. Changes in masticatory movements can have repercussions on quality of life because people avoid certain foods if they are unable to chew them, or swallow the food without it being properly crushed [5]. This is more notorious in older people [6]. The analysis of masticatory characteristics can be used to design specific diets [7]. Hence, it is of interest to analyze movement patterns during mastication.

Various recording methods have been proposed to analyze mandibular movements during mastication. Video recording has been used as a simple method to analyze mastication by counting the number of masticatory cycles, the duration of each cycle and the duration of complete mastication [8,9]. Here, the subject is filmed during mastication and later, an observer determines the chewing strokes and the total duration of every cycle with a chronometer. The use of external markers to analyze movement is a technique that can describe mandibular movement using spatial coordinates, recording the trajectory of these markers and analyzing them using software (Optotrak®, Northern Digital, Waterloo, ON, Canada) [10]. These are mounted on structures fixed to the mandible, commonly on the lower incisors [11,12,13]. This technology makes kinematic analysis possible, since by obtaining the coordinates of the markers, it is possible to know the displacement, speed and acceleration of the mandible in three dimensions [14]. Another method to record three-dimensional movement during mastication is using a kinesiograph [15], which consists of a facebow, where the sensors are mounted and a magnet is adhered to the vestibular part of the lower incisors [16]. The sensors measure the variation in the magnetic field of the magnet and its position is calculated using software (K5−R Mandibular Kinesiograph, Myo-Tronics Research Inc., Seattle, WA, USA) [17]. The electromagnetic articulograph uses a similar principle. It uses electromagnetic waves that induce currents in small coils that act as sensors. Depending on the distance between the receiver coil and the emitter coil, the induced current intensity will vary. Based on this, the equipment determines the position of the coil within the measurement area [18]. The sensors are attached to the patient with tissue adhesive [19].

This work discusses the methods developed to analyze mandibular movement in the Oral Physiology Laboratory of the Dental Sciences Research Center (Centro de Investigación en Ciencias Odontológicas—CICO), in the Faculty of Dentistry at the Universidad de La Frontera (Temuco, Chile) using electromagnetic articulography with the AG501 3D EMA articulograph (Carstens Medizinelektronik, Lenglern, Germany) and MATLAB^®^ routines (R2020a, version 9.8.0, The MathWorks Inc., Natick, MA, USA). In the following paragraphs, EMA will be described in detail together with these protocols and compared to other devices used to analyze the same parameters. This will show that electromagnetic articulography can encompass various aspects of the analysis of mandibular movement that usually are covered using different devices and cover the limitations present in other recording devices.

## 2. Electromagnetic Articulography

Electromagnetic articulography (EMA) is a recording technique based on variable magnetic fields that tracks the movement of points inside and outside the oral cavity [20]. This consists of magnetic field transmitter coils that induce currents on small receiver coils located at the points of interest. Each transmitter coil generates a magnetic field at different frequencies in order of KHz. Depending on the distance of the receiver coil from each transmitter coil, the induced current will have different frequency components. The distance to each emitter coil can be determined via post-processing, and the position of the receiver coil can be determined using triangulation [21]. At first, this technology was limited to 2D analysis in the sagittal plane [22], but the latest developments allow for 3D analysis [23]. A consideration to keep in mind about the use of articulography is that this technique is sensitive to the presence of metallic objects or proximity between sensors (e.g., the AG501 articulograph manual indicates that the sensors must be at least 8 mm apart).

Unlike other methods (ultrasound, kinematography), electromagnetic articulography records the movement of several articulators simultaneously, and is biologically safe and minimally invasive [24]. When the sensors are placed in the mouth, patients have an adaptation time of approximately 10 min [25].

Articulography devices are safe for health, complying with various standards regarding exposure to magnetic fields [26]. However, there are certain considerations with regard to patients who have implanted devices such as pacemakers [27], cochlear implants [28] or insulin pumps [29], because the electromagnetic field can affect their correct operation. Joglar et al. [30] conducted tests with several pacemakers and defibrillators with the AG100 articulograph, finding them compatible with the AG100. Katz et al. [31] tested the compatibility of the Clarion 1.2 series of cochlear implants with the AG 100 and found no adverse effects on the functioning of the implant or on the user’s perception of speech. Mücke et al. [32] analyzed patients with essential tremors undergoing treatments with deep brain stimulation (DBS) of the thalamus. The participants were studied using the AG501, with no adverse effects reported. Some studies warn about exposure for pregnant women due to the effects of the electromagnetic field not being clear, which is why it is preferable to avoid risks [33].

## 3. AG 501 Articulograph

**Figure 1:**
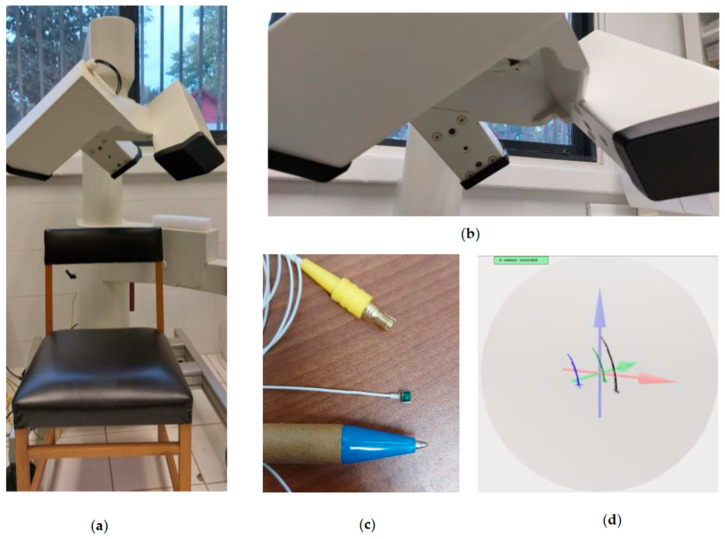
(**a**) AG501 electromagnetic articulography; (**b**) emitter coils; (**c**) sensor coil; (**d**) measurement area.

The AG 501 electromagnetic articulograph is a device developed by Carstens (Bovenden, Germany), which enables the three-dimensional study of the movement of the structure to which the sensors are adhered [24] (Figure 1a). It has nine emitter coils that generate electromagnetic fields in a frequency range of 7.5 to 13.75 KHz (Figure 1b). It can use up to 16 sensors, formed by small coils adhered to the mobile element to be studied using tissue adhesive (Figure 1c). The subject sits with their head within the measurement area, which is a sphere of 30 cm in diameter (Figure 1d) [34]. The red axis is the *x*-axis, the green one is the *y*-axis and the blue one is the *z*-axis. The AG501 has a sampling frequency of 1250 Hz [35] and an accuracy of 0.3 mm [36]. These characteristics are sufficient to record the movements of articulators such as the tongue, the lips or the mandible [37].

This device is certified by the Federal Communications Commission (an independent US government agency) as a low-power transmission device. This range is smaller than the frequency range of radio transmission devices like cell phones (10 MHz to 300 GHz) [38].

### 3.1. Head Correction

**Figure 2:**
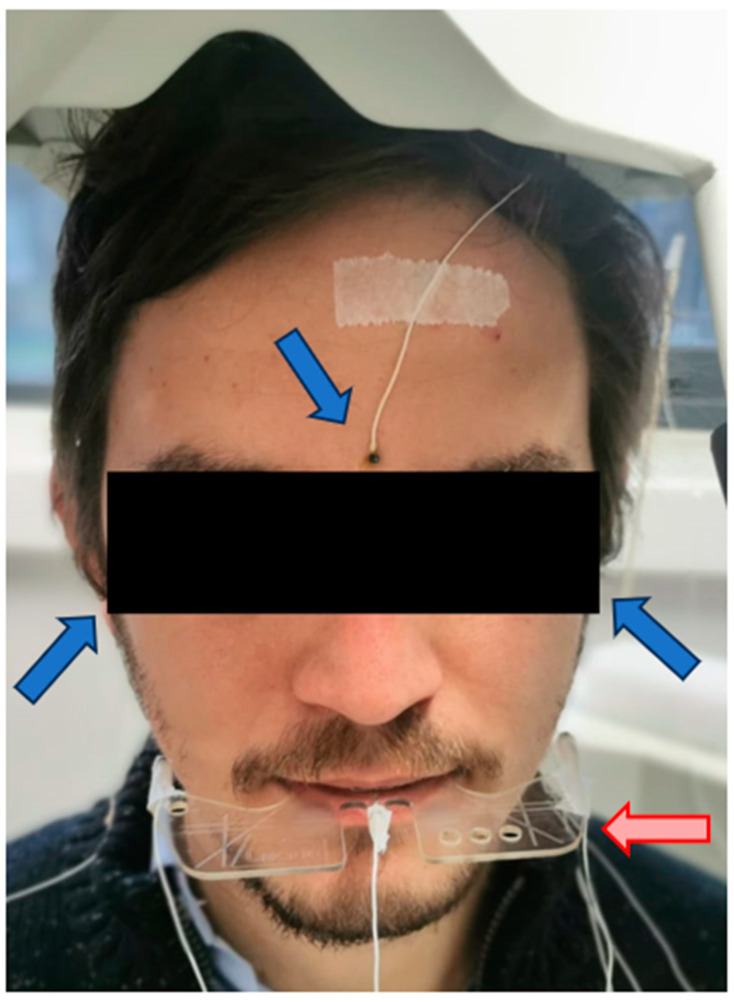
Configuration of the sensors. The reference sensors, glabella and right and left mastoids are indicated in blue. The bite plane device is in red.

**Figure 3:**
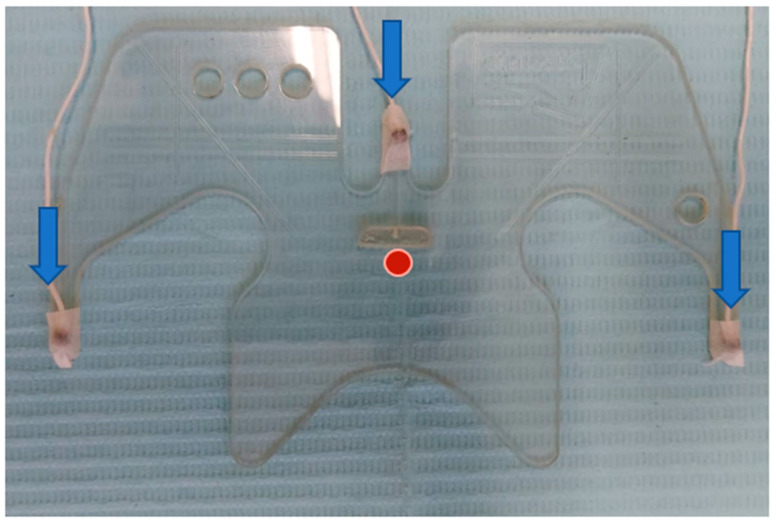
The sensors mounted on the bite plane are in blue. The point where the coordinate origin is established is in red.

The AG 501 can correct the movements and the inclination of the head to obtain the absolute movements of the mandible, compared to a system generated by reference sensors. This procedure is called Head Correction. For this, three reference sensors are placed at points that are independent of mandibular movement; for example, the cutaneous points of the right and left mastoids, process and glabella [39] (Figure 2). In addition, the device has an accessory, called the bite plane, which aligns the horizontal plane of the system with the occlusal plane of the patient. This eliminates possible parallax errors and locates the origin of the system in the occlusal plane at the midline of the upper incisors on the vestibular side [40]. This is a support that accommodates three sensors that remain outside the mouth in a fixed configuration. The participant is asked to hold the bite plane, with the incisors immediately behind the stop in the center of the bite plane in front of the central sensor (Figure 3). With the three reference sensors and the three bite plane sensors, a first recording is conducted to perform the head correction. From this recording, the others are corrected to put them in line with the reference system.

### 3.2. Data Acquisition

The AG501 generates binary files, with three-dimensional coordinates of the active sensors given in millimeters. These can be extracted and processed using routines specifically developed for this purpose. Given that the masticatory movement is to be analyzed, the recording sensor must be placed at some point on the mandible. Typically, it is placed on the gums, underneath the interincisal line on the vestibular side [34].

## 4. Border Movements

There are limits to the movements that the mandible can perform. These are defined by the border movements on the sagittal, coronal and horizontal planes [41]. To describe the border movements, the participant is asked to place the mandible in extreme positions on each plane, describing Posselt’s envelope of motion [42]. Border movements tend to be used as a clinical tool to evaluate the state of the masticatory system [43]. A limitation of mandibular border movement is a symptom of temporomandibular joint disorder [44]. Yu et al. [45] analyzed the border movements in patients undergoing orthodontic treatment, observing a greater range after the treatment.

**Figure 4:**
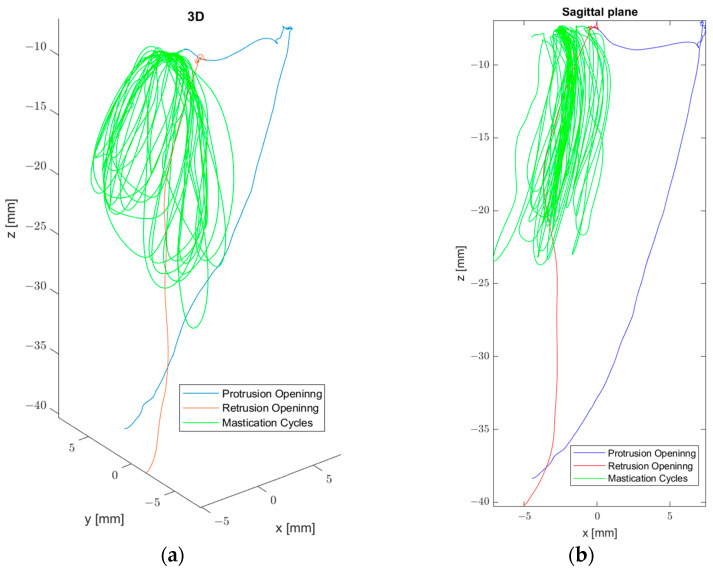
(**a**) Sagittal view of Posselt’s envelope of motion, 3D view. (**b**) Lateral view.

Fuentes et al. [38] developed a protocol to record Posselt’s envelope of motion. The sensor was placed on the gum on the vestibular side of the lower incisors, on the interincisal line. The patient was asked to perform border movements in the sagittal and coronal plane. In Figure 4, the Sagittal view of Posselt’s envelope of motion can be seen.

### Area of Posselt’s Envelope of Motion

Once Posselt’s envelope of motion has been defined, it can be analyzed to extract characteristics to compare the study groups.

**Figure 5:**
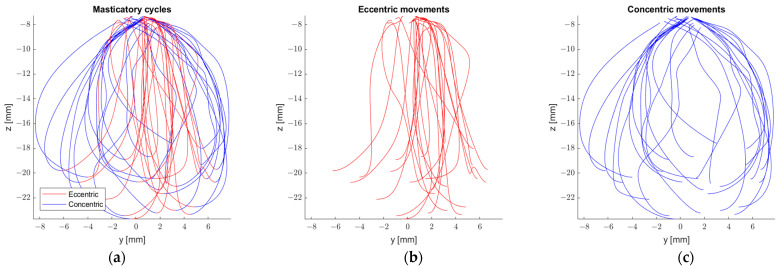
(**a**) Masticatory cycles. (**b**) Eccentric movements. (**c**) Concentric movements.

Chuhuaicura et al. [46] in their study on eccentric and concentric border movements, found significant differences in the described area concentrically and eccentrically from the frontal and sagittal polygons, with the area being greater concentrically in both cases. In this study, subjects were asked to perform border movements in the traditional way [41] and then in reverse, with a movement sensor attached to the interincisal line on the lower incisors. Based on these movements, the polygon area was calculated with a Matlab function, polyarea, with the coordinates of the movement sensor as an input. After performing the border movements, the subjects performed peanut chewing. With the same procedure, the area of each chewing cycle was obtained and compared to the area obtained for the border movements. Chuhuaicura et al. found that for the frontal polygon, the chewing area is around 10% of the frontal border movement area, for sagittal it is around 5% and for horizontal it is around 15%. Figure 5 provides an example of eccentric and concentric movements during mastication.

## 5. Kinematic Analysis

It is possible to perform kinematic analyses with sensor coordinates. If we define the position of sensor S as:

(1) $$P_{\left[n\right]}=(x_{n},y_{n},z_{n})$$

We can define the speed and acceleration of this point as [47]:

(2) $${\bar{v}}_{\left[n\right]}=\frac{\bar{P_{\left[n+1\right]}-P_{\left[n\right]}}}{∆t}=\langle x_{n+1}-x_{n},\;\;\;\;\;y_{n+1}-y,\;\;\;\;\;z_{n+1}-z_{n}\rangle \frac{1}{∆t}=\langle v_{xn},\;\;\;\;\;v_{yn},\;\;\;\;\;v_{zn}\rangle$$

(3) $${\bar{a}}_{\left[n\right]}=\frac{{\bar{v}}_{n+1}-{\bar{v}}_{n}}{∆t}=\langle v_{xn+1}-v_{xn},\;\;\;\;\;v_{yn+1}-v_{n},\;\;\;\;\;v_{zn+1}-v_{zn}\rangle \frac{1}{∆t}=\langle a_{xn},\;\;\;\;\;a_{yn},\;\;\;\;\;a_{zn}\rangle$$

where *n* indicates the number of the sample within the file generated by the AG501. Δ*t* will be determined by the sampling frequency (fs) of the device, 1250 Hz, for which Equations (2) and (3) can be re-written as:

(4) $${\bar{v}}_{\left[n\right]}=\langle x_{n+1}-x_{n},\;\;\;\;\;y_{n+1}-y,\;\;\;\;\;z_{n+1}-z_{n}\rangle f_{m}=\langle v_{xn},\;\;\;\;\;v_{yn},\;\;\;\;\;v_{zn}\rangle$$

(5) $${\bar{a}}_{\left[n\right]}=\langle v_{xn+1}-v_{xn},\;\;\;\;\;v_{yn+1}-v_{n},\;\;\;\;\;v_{zn+1}-v_{zn}\rangle f_{m}=\langle a_{xn},\;\;\;\;\;a_{yn},\;\;\;\;\;a_{zn}\rangle$$

Patients with a temporomandibular joint disorder show a lower mastication speed than healthy subjects [48,49,50]. In line with these observations, Bakke et al. [51] found that patients with unilateral pain in the temporomandibular joint showed an increase in mastication speed after undergoing treatment.

## 6. Masticatory Cycles

**Figure 6:**
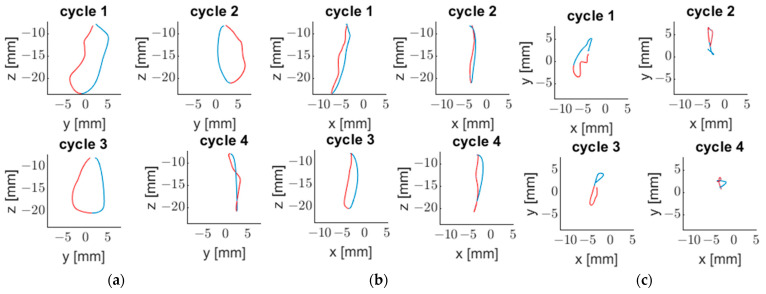
Masticatory cycles corresponding to a full recording of peanut mastication. (**a**) Frontal plane. (**b**) Sagittal plane. (**c**) Horizontal plane. The mandibular descent is in blue, and the ascent is in red.

Vargas-Arguto et al. [52] managed to differentiate each masticatory cycle using Matlab routines (Figure 6).

Eleven healthy participants (five men and six women) with a full denture up to the first molar of each hemiarch were included. They were asked to chew 3.7 g of peanut and carrot disks (1 × 2 cm) until they had the need to swallow. Three records were made for each food. Chewing cycles were identified using the vertical position of the sensor. Using a threshold value, the beginning and the end of each cycle was established. With the masticatory cycles defined and the previously mentioned tools, each cycle can be analyzed in isolation. Using Matlab routines, Vargas-Arguto et al. [52] identified the number of cycles, the masticatory cycle frequency, the speed of the ascent and descent of each cycle, using the Section 5 equations, and the area of each, using the same method as Chuhuaicura et al. [46]. They noted that men presented a greater masticatory frequency and mandibular speed than women. Only significant differences in the horizontal polygon area between peanut and carrot chewing were found.

The study of movement patterns is relevant to understanding the neural control of mastication and for denture design [53]. In their study, Lepley et al. [54] found that patients with poor masticatory performance presented irregular patterns and that they differed from those with greater masticatory efficiency. Flores-Orozco et al. [55] found that a large mandibular opening and a small angle of lateral excursion are associated with greater masticatory efficiency.

Mastication patterns can be divided into four types: alternating bilateral, simultaneous bilateral, unilateral preference and chronic unilateral [56]. Several studies have endeavored to identify factors that determine the preference for a mastication side. Christensen & Radue [57] analyzed the relation between the side of mastication preference and the dominant hand, but did not find a significant relation. García et al. [58] reported that the choice of one of these patterns is influenced by the consistency of the food. Paphangkorakit et al. [59] indicated that when a concerted effort is required to cut and grind the food, subjects have a side of preference (for example with pork), whereas with more fragile foods (almonds) they tend toward bilateral mastication. Hannam et al. [60] suggested that the preference for a side may be influenced by the ability to place the mandible on that side.

## 7. Analysis of Mouth Opening

Mouth opening is an important indication of the functionality of the temporomandibular joint (TMJ) [61]. Marinelli et al. [40] designed a protocol to analyze mouth opening using EMA by using four parameters: vertical distance, Euclidean distance, trajectory and angle. This protocol was tested over a mandibular phantom, with movement sensors placed on the interincisal line between the first molar and last premolar on the left and right. With the sensors placed, four openings were performed, simulating 1, 2, 3 and 4 cm of mouth opening from the maximum intercuspation position (MIP) in vertical distance.

Vertical distance was obtained via Equation (6):

(6) $${\;dz}_{j}=z_{j}-z_{PMI}$$

where *z_j_* is the vertical position of the interincisal sensor at each opening and *z_PMI_* is the vertical position of the sensor with the phantom closed.

Euclidean distance is the distance between the reached position and MIP:

(7) $$d_{j}=\sqrt{{\left(x_{j}-x_{PMI}\right)}^{2}+{\left(y_{j}-y_{PMI}\right)}^{2}+{\left(z_{j}-z_{PMI}\right)}^{2}}$$

Trajectory is the length of the path traveled by the sensor during the opening:

(8) $$T=\sum_{i=1}^{N-1}\sqrt{{\left(x_{i+1}-x_{i}\right)}^{2}+{\left(y_{i+1}-y_{i}\right)}^{2}+{\left(z_{i+1}-z_{i}\right)}^{2}}$$

For the opening angle, the data in the premolar and interincisal sensors were used. In the first place, two vectors were defined.

(9) $$\bar{V_{R}}=\bar{S_{2}S_{1}}=\langle x_{2}-x_{1},y_{2}-y_{1},z_{2}-z_{1}\rangle$$

(10) $$\bar{V_{L}}=\bar{S_{3}S_{1}}=\langle x_{3}-x_{1},y_{3}-y_{1},z_{3}-z_{1}\rangle$$

where *S*_1_, *S*_2_ and *S*_3_ are the interincisal, right premolar and left premolar sensors, respectively. With these two vectors, the perpendicular vector was calculated based on the cross product.

(11) $$\bar{V_{P}}=\bar{V_{L}}\times \bar{V_{R}}$$

With this vector defined, the inclination angle can be calculated for every opening with respect to MIP as follows:

(12) $$\theta_{j}=\left|\cos^{-1}\left(\frac{\bar{V_{PMI}}\cdot \bar{V_{Pj}}}{\left|\bar{V_{PMI}}\right|\left|\bar{V_{Pj}}\right|}\right)\right|$$

In this research, the openings achieved according to Equation (6) were 9.8, 20.2, 30.4 and 38.9 mm. In concordance with this, the Euclidean distances were 10.2, 21.5, 33.0 and 43.1 mm and the trajectories were 16.7, 28.1, 39 and 48.7 mm, respectively. The opening angles were 5.6, 12, 18.3 and 23.8 degrees, respectively.

Mouth opening has been used to assess the effect of surgical procedures [62] and the efficacy of anti-inflammatory drugs used in postoperative surgical removals [63].

## 8. Electromyography

Gómez et al. [64] reported a protocol to simultaneously record electromyography (EMG) and electromagnetic articulography, which together with the previously presented variables provides a comprehensive examination of masticatory function. In a recent work, Lezcano et al. [65] reported a recording protocol in which they synchronized the recordings of EMA and EMG.

Ranges of motion are influenced by the muscles of mastication [66]. As mentioned previously, mastication is a process coordinated by the centers of the brainstem, which receive feedback from a set of sensory receptors. The texture of the food influences the force, the duration of the masticatory cycle and the number of cycles before swallowing [67]. Some studies have found a link between the characteristics of the food and the electromyographic activity of the elevator muscles during mastication [68,69,70,71]. The level of dentition, age [72] or the presence of temporomandibular joint disorders [73,74] also affect electromyographic activity.

## 9. Discussion

As previously described, the analysis of masticatory movement is of great interest for both understanding masticatory function and for the diagnosis of disorders or assessing the effectiveness of a treatment.

In their works on border movements, Kataoka et al. [44] and Yu et al. [45] used a video system to record mandibular movement. Kataoka used a system based on LEDs and a facebow, while Yu used two video cameras placed in front of and beside the patient with a 90° angle between them. Two resin reference balls were used to label the cutting edges between the nose and the center point of the mandibular incisors. The method presented by Yu is simple; however, it lacks a head correction procedure, and the markers are placed on the patient’s skin, which may move during chewing. Kataoka makes use of a facebow which enables head correction, but it could result in unnatural chewing. Ferrario et al. [11] avoid both problems using reference markers and six high-resolution cameras. The movement was recorded using three extraoral markers placed on an equilateral triangular antenna, anchored on the mandibular anterior gingival line just out of dental contact. However, due to this arrangement, the antenna may be displaced by the lips, making it difficult to capture natural movements during chewing. Lepley et al. [54] use the Optotrak 3020^®^ (Northern Digital, Waterloo, ON, Canada) system with a single diode attached to the subjects’ chin and glasses with a diode arrangement to correct for head movement. This system covers the previously mentioned limitations but needs a 2 m space between the subject and the cameras. Also, the movement marker is attached to the patient’s skin.

The ARCUSdigma device is considered the gold standard for recording mandibular movement [75]. It is based on the use of an ultrasound, with emitters mounted on a facebow fixed to the mandible [76]. Sójka et al. [76] used this device to evaluate mandibular movement. Given that the device must be placed inside the mandible, this interferes with natural movement during mastication. An advantage of this system is the possibilty to record condylar movement [77].

A kinesiograph also uses a facebow with a magnet attached to the incisors. This device senses the maginetic field of the magnet to record movement. It has been used to describe mandibular movement [78] and Posselt polygons [79]. However, this device has been questioned due to inconsistent results [80].

In previous paragraphs, we described parameters used to study chewing and devices designed to achieve this. Posselt polygons, kinematic analysis, mouth opening and electromiographic activity [81,82,83] have been the objects of several studies. These variables have been analyzed with different devices to try to overcome certain problems that arise when recording mandibular movement, head movement, faithful tracking of mandibular movement and modifying the natural movement during chewing. Border movements have been analyzed using EMA in several studies [19,38], and a new variable has been introduced by analyzing border movements eccentrically and concentrically [46], as well as through symmetry analysis [84]. In addition, it has been made possible to analyze masticatory kinematics, differentiation of each cycle and its stage of ascent and descent in the area described [52].

Regarding sensor placement, these are attached to the gums to avoid skin movement. Due to their dimensions, they do not interfere with movement, and head movement can be filtered using the head correction procedure. Three-dimensional electromagnetic articulography allows for analysis of reliable accuracy [37].

Future studies should involve groups of greater diversity, with different skeletal classes or that suffer from a temporomandibular joint disorders. In addition, studies can be carried out on patients who are undergoing treatment and observe whether there are significant differences between the variables that are analyzed before and after the treatment. The protocol for the use of EMA in conjunction with EMG was made by analyzing the vertical dimension statically, which is why a future study should be conducted where electromyographic activity is recorded during masticatory movements.

An obstacle to the use of EMA is the interference of metallic elements with the magnetic fields, which is why the analysis of subjects with metallic prostheses is limited. This issue is also shared with the kinesiograph. In addition, the sensors must be 8 mm apart so as not to interfere with each other [24]. A disadvantage with respect to the ARCUSdigma device is the posibility of measuring condylar movement. Due to its costs and complexity, EMA is limited to research use only.

## 10. Conclusions

Electromagnetic articulography is a tool that allows for complete analysis of the various aspects used to evaluate movement during mastication, along with EMG. The differentiation of each cycle makes it possible to visualize the change in mastication patterns as the food is crushed and forms an alimentary bolus. In addition, it allows the analysis of new variables, such as the frequency of cycles, speed of ascent and descent and the area of each cycle. Several studies have already been conducted with this technology, but more analyses are needed with a greater diversity of subjects and cases.

## 11. Future Directions

The use of EMA and data postprocessing offers new possibilities in the assessment of functional movements of mastication, and combined with the EMG it is possible to cover all aspects of past research, like mouth opening, preference side, number of cycles, kinematic analysis, etc., with a unique technique. However, a new paradigm can be introduced in the analysis of masticatory movement as the normalization of both mandibular opening, area description and EMG activity. Normalization is a technique that could allow for comparison of parameters between patients, avoiding differences due to body size, age or gender. This can be conducted cycle by cycle since individualization is possible. With these possibilities, a standardization of mandibular movement records and variables of interest can be performed to avoid differences between studies due to different recording methods or devices. A potential challenge in using EMA in the field of dentistry is that the metal components of prosthetics can interfere with the equipment. A deep study of the presence of metal elements and their interference with EMA must be conducted.

## Data Availability

The data presented in this study are available on request from the corresponding author. The data are not publicly available due to no public database is available.
